# Development of a percutaneous coronary intervention patient level composite measure for a clinical quality registry

**DOI:** 10.1186/s12913-019-4814-6

**Published:** 2020-01-17

**Authors:** Darshini Ayton, Sze-Ee Soh, Renata Morello, Susannah Ahern, Arul Earnest, Angela Brennan, Jeffrey Lefkovits, Susan Evans, Christopher Reid, Rasa Ruseckaite, John McNeil

**Affiliations:** 1 Department of Epidemiology and Preventive Medicine, Monash University, Melbourne, Australia; 2 Department of Physiotherapy, Monash University, Melbourne, Australia; 3 NHMRC Centre for Research Excellence in Cardiovascular Outcomes Improvement, Curtin University, Bentley, Western Australia Australia

**Keywords:** Composite measure, Patient reported outcome, Clinical outcome, Percutaneous coronary intervention

## Abstract

**Background:**

Composite measures combine data to provide a comprehensive view of patient outcomes. Despite composite measures being a valuable tool to assess post-intervention outcomes, the patient perspective is often missing. The purpose of this study was to develop a composite measure for an established cardiac outcome registry, by combining clinical outcomes following percutaneous coronary interventions (PCI) with a patient-reported outcome measure (PROM) developed specifically for this population (MC-PROM).

**Methods:**

Two studies were undertaken. Study 1: Patients who had undergone a PCI at one of the three participating registry hospital sites completed the 5-item MC-PROM. Clinical outcome data for the patients (e.g. death, myocardial infarction, repeat vascularisation, new bleeding event) were collected 30 days post-intervention as part of routine data collection for the cardiac registry. Exploratory factor analysis of clinical outcomes and MC-PROM data was conducted to determine the minimum number of constructs to be included in a composite measure. Study 2: Clinical experts participated in a Delphi technique, consisting of three rounds of online surveys, to determine the clinical outcomes to be included and the weighting of the clinical outcomes and MC-PROM score for the composite measure.

**Results:**

Study 1: Routine clinical outcomes and the MC-PROM data were collected from 266 patients 30 days post PCI. The MC-PROM score was not significantly correlated with any clinical outcomes.

Study 2: There was a relatively consistent approach to the weighting of the clinical outcomes and MC-PROM items by the expert panel (*n* = 18) across the three surveys with the exception of the clinical outcome of ‘*deceased at 30 days’*. The final composite measure included five clinical outcomes within 30 days weighted at 90% (new heart failure, new myocardial infarction, new stent thrombosis, major bleeding event, new stroke, unplanned cardiac rehospitalisation) and the MC-PROM score (comprising 10% of the total weighting).

**Conclusions:**

A single patient level composite score, which incorporates weighted clinical outcomes and a PROM was developed. This composite score provides a more comprehensive reported measure of individual patient wellbeing at 30 days post their PCI-procedure, and may assist clinicians to further assess and address patient level factors that potentially impact on clinical recovery.

## Background

As a result of advances in interventions, mortality rates in cardiac patients have declined over the past decades and more people live longer with heart disease [1]. These patients are at high risk of experiencing a recurrent cardiac event and the focus is now on optimising the quality of secondary preventive care [2]. In a scientific statement, the American Heart Association states that implementation of patient reported outcome measures (PROMs) in clinical settings has “the potential to support clinical care, evaluate healthcare quality, quantify an important component of procedural appropriateness, identify patients for prognostic discussions and serve as a foundation for shared medical decision making” [3]. In surveillance, PROMs have the potential to quantify the impact of the disease as well as of the therapies and interventions for these conditions on patients’ lives [3].

Clinical quality registries (CQR) systematically monitor the quality of health care within specific clinical domains by routinely collecting, analysing and reporting health-related information [4]. The increasing number of CQRs is in large part due to their unique ability to provide clinicians and administrators with regular feedback about clinical performance (including outcomes) that cannot be provided by other strategies such as individual hospital datasets. The information that CQRs generate is respected by clinicians and has the credibility to drive change. In doing so, registries complement a variety of other approaches to clinical quality improvement such as sentinel event reporting, limited adverse occurrence screening, incident reporting, morbidity and mortality reviews and patient satisfaction surveys [5–7]. By implementing PROMs, registries can provide a more comprehensive measure of procedure outcomes and contribute to surveillance, enable benchmarking and drive quality improvement of health care services [8].

### Composite measures for reporting outcomes

A composite measure is a summary variable created by grouping two or more outcomes which are related to one another conceptually or statistically [9]. Composite measures facilitate benchmarking performance and encourage quality improvement initiatives across various industry sectors and organisations [10, 11]. Composite measures assist in health related decision making, evaluation of an individual’s health outcomes, and assessment of standards, performance and quality of care across different health sectors [12–16]. Composite measures may also be easier for clinicians to operationalise as they capture both the good and poor outcomes of care in one measure.

Composite measures have typically been derived from combining clinical outcomes, with a focus on clinician and healthcare facility performance as an outcome**.** For example, the Coronary Artery Bypass Graft (CABG) Composite Score from the Society of Thoracic Surgeons CABG registry is calculated using a combination of 11 clinical outcomes. The composite score is divided into four domains: mortality, morbidity, use of internal mammary artery, and perioperative medication [17]. This score provides an indicator of surgeon performance. Missing from this measure, and other cardiac outcome composite measures, is the patients’ perspective of recovery that incorporates quality of life and functioning post-procedure, which may be predictive of longer-term clinical outcomes [2]. Patient pathways following percutaneous coronary intervention (PCI) have traditionally focused on treatment efficacy and safety, with a consequent lack of emphasis on PROMs in recent PCI and Acute Coronary Syndrome (ACS) guidelines.. As an example, the most recent Australian ACS guidelines does not list a patient reported outcome measure (PROM) in the section on Measures of Performance and Clinical Standards [18].

The Victorian Cardiac Outcomes Registry (VCOR) is a CQR that provides performance outcomes for PCI at a health service level. The registry incorporates all 31 hospitals that perform PCI in the state of Victoria, Australia and is now well-established, with five years of data collection and over 40,000 case records. From 2019, VCOR will provide benchmarked reports to the Victorian Department of Health and Human Services to include in their state-wide quality reporting framework.

**Fig. 1:**
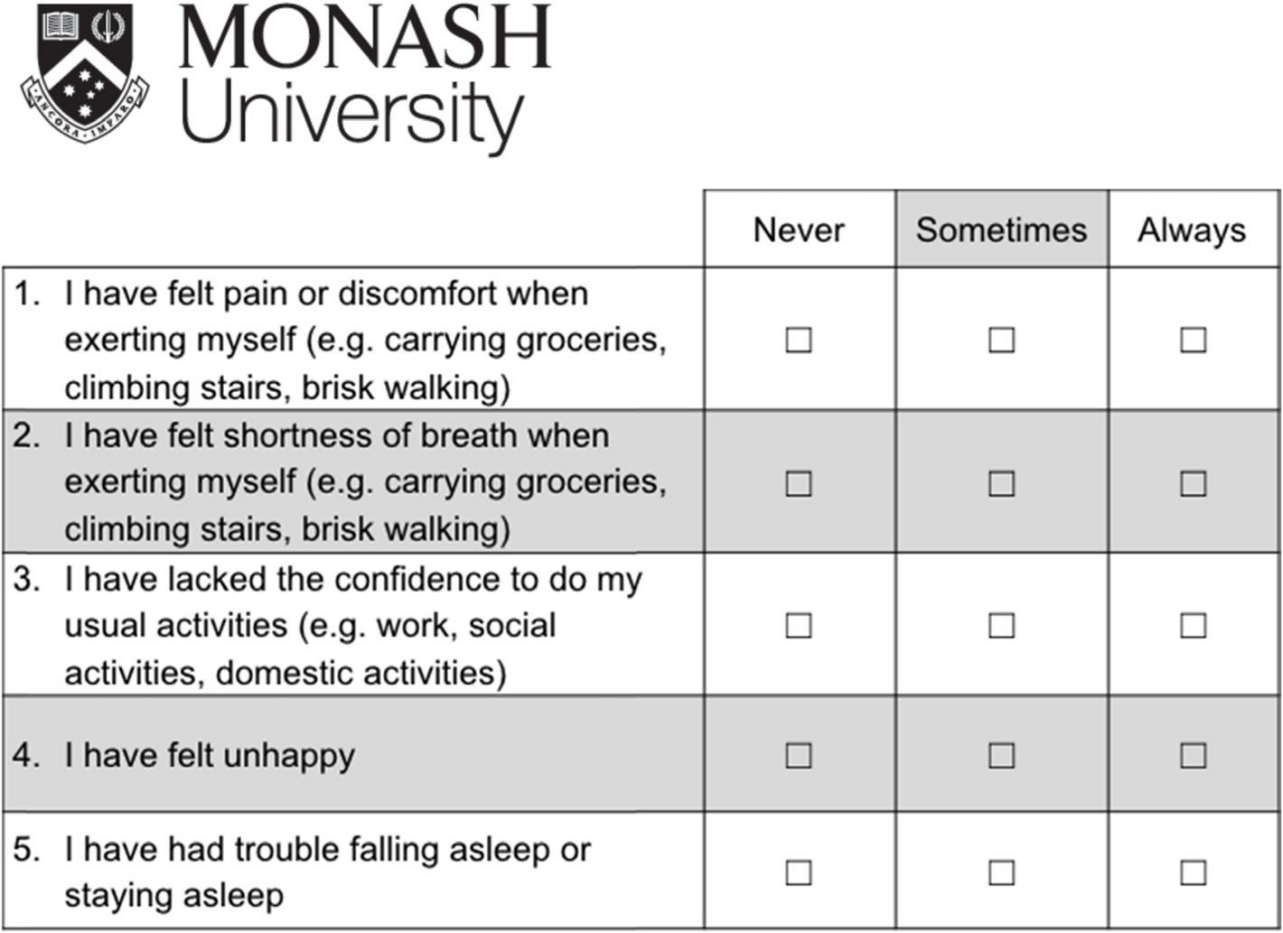
MC-PROM

A PROM has been developed for use by VCOR to assess recovery following PCI at 30 days post procedure [19–21]. The Monash University Cardiac PROM (MC-PROM) was developed via a three-stage research project that involved patients in every aspect. Stage one consisted of focus groups and interviews with 32 patients who had a PCI in the preceding six months. Patients were asked to identify physical, psychological and functional outcomes they perceived as important in terms of recovery from their PCI [19]. Based on this stage, 10 outcomes were identified. In stage two, a discrete choice experiment survey with 138 patients within six months of their PCI was conducted to establish patient preferences for the 10 outcomes. The perceived important PCI outcomes were reduced to eight after this stage [20]. The final stage was to identify and validate the best set of items to form a concise and psychometrically sound PROM using Rasch analysis. A consecutive sample of 200 patients participated in a telephone survey 30 days following their PCI procedure. Five items were identified that can be included in a PROM post-PCI (Fig. 1). The MC-PROM was found to have good internal construct validity and acceptable internal consistency reliability [21]. We found evidence to sum scores from each item to obtain an overall score scored out of 10, with lower scores indicating better patient recovery [21]. The MC-PROM was also found to have a moderately strong and negative correlation with the EQ-5D utility score (Spearman’s *rho* − 0.53; *p* < 0.01).

The aim of this project was to develop a composite measure that combines clinical outcomes with the MC-PROM to measure the quality of PCI at 30 days in a cardiac registry. The specific objectives were to:

1. Determine the minimum number of clinical outcomes to be included in the composite measure;
2. Assign weightings to the outcomes in the composite measure; and,
3. Propose possible end points for the future validation of the composite measure.

## Methods

**Fig. 2:**
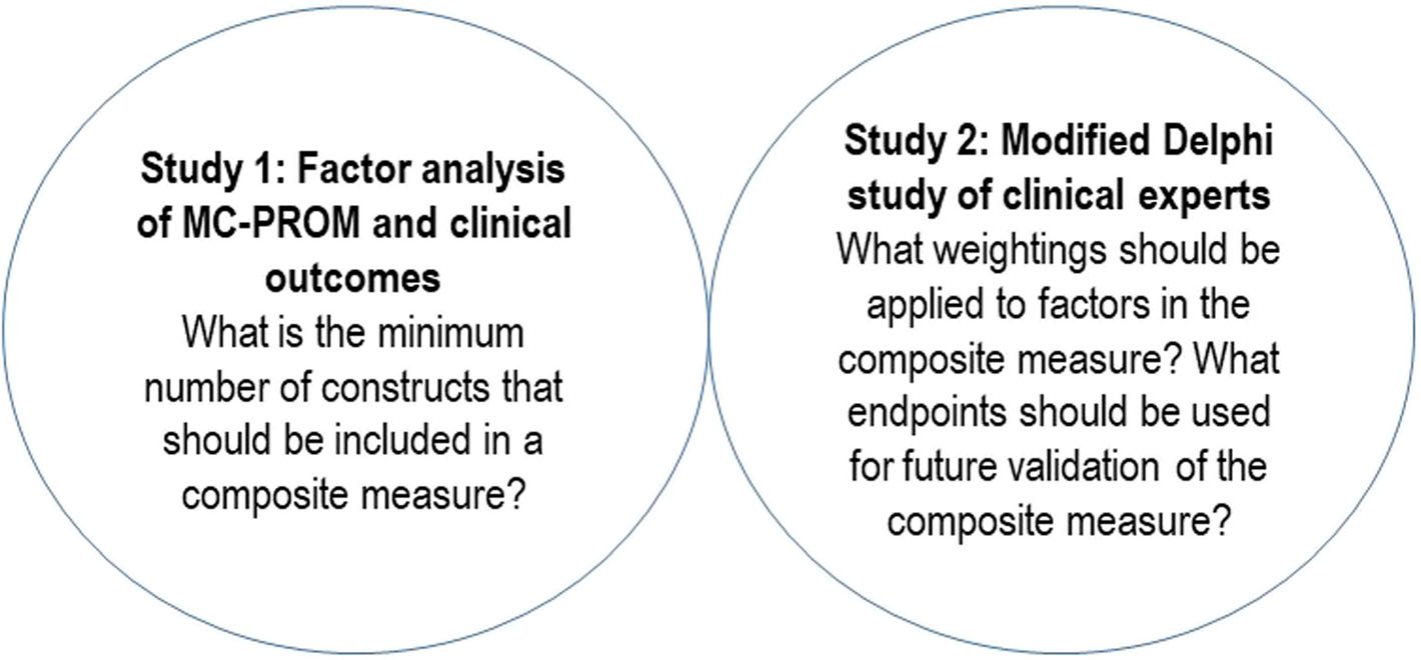
Overview of project studies

This project consisted of two studies (Fig. 2) to address the objectives described above.

### Study 1: factor analysis of MC-PROM and clinical outcomes

This study was designed to address objective 1 – to determine the minimum number of clinical outcomes to include in the composite measure.

#### Participants

Three tertiary public hospitals in metropolitan Victoria, Australia that have contributed to VCOR since 2013 were invited to participate in this study. The hospitals ranged in size from moderate (400–600 beds) to large (> 600 beds). Patients who were 18 years of age or older, and had a PCI at any of these participating hospitals were invited to complete the MC-PROM during their 30-day follow-up (administered via telephone) by the hospital between September 2017 and February 2018. Verbal consent was obtained from patients. The following script was administered by the facility VCOR data managers: *“We are trialling some new questions to find out about your health and wellbeing following your PCI. These questions were developed by patients and researchers. I will ask you five questions that relate to how you have felt in the last seven days. Are you happy for me to ask you these questions?”* If the patient declined, the data manager did not ask the PROM questions.

#### Data collection

Participating patients were asked to provide responses to the MC-PROM by the VCOR data manager during their routine 30-day follow up phone call. Demographic information and data regarding the seven clinical outcomes were obtained from data routinely collected by VCOR that is collected and managed using REDCap electronic data capture tools hosted at Monash University [22]. Data collection was from the 10th of September 2017 to the 15th of February 2018.

#### Data analysis

Descriptive statistics were used to profile the cohort, the clinical outcomes and MC-PROM data collected by VCOR. Exploratory analysis was conducted to determine the minimum number of constructs that could be included in a composite measure for patients following a PCI. Principal component analysis was used to extract the factors and explore the underlying structure of the composite measure. The Kaiser’s criterion, where all outcomes with an eigenvalue of 1 or more, in conjunction with the Scree test, determined the number of outcomes (factors or dimensions) to be retained. Factor loadings were also used to weight the different clinical outcomes. There is no standard method for estimating sample size in factor analysis, although it is generally recommended that the number of observations is dependent on the number of outcomes that will be examined in the model. Traditional recommendations within psychometrics is to include at least 10 to 20 cases per variable to allow significant testing of model effects [23]. We anticipated that the composite measure would include 8–10 outcomes when combining the available clinical indicators with the MC-PROM, hence a sample size of between 80 to 160 participants was required for this analysis. All statistical analyses were conducted using SPSS Version 25 (IBM Corp, IBM Statistics for Windows. Armonk, NY: IBM Corp).

### Study 2: modified Delphi to determine the weighting of the factors in the composite measure and possible end points for future validation studies

This study was designed to address objectives 2 and 3:

2. Assign weightings to the outcomes in the composite measure; and,
3. Propose possible end points for the future validation of the composite measure.

#### Participants

Convenience and purposive sampling (to ensure a mix of metropolitan and regional, and public and private settings) was adopted to recruit clinical experts to the Delphi study. The Clinical Director (author JL) invited cardiac clinicians, nurses, VCOR representatives and health service managers to participate in the Delphi. As the final composite measure will be used by VCOR and cardiac clinical experts, participants for this study focused on ensuring representation from this group. Invitations to participate were sent via the VCOR clinical director as a personal email with a survey link. Consent was implied by survey completion. The surveys were hosted online via the survey platform Qualtrics (Qualtrics, 2018). To maintain the confidentiality of the participants, no demographic or professional information was collected in the survey.

#### Data collection

In the first round, participants had two tasks. First they were asked to rate the importance of each of the seven clinical outcomes (Table 1) and the MC-PROM items on a scale of 1 to 9 (9 = highly important, 1 = not important) based on whether it represents quality of care. Participants were asked to provide a rationale for their rating.

**Table 1:** Characteristics of patients following PCI procedure

	Patients in this study (*n* = 266)	
Male, *n* (%)	212	(79.7)
Age, mean (SD)	64.3	(11.0)
Clinical presentation, *n* (%)		
STEMI	95	(35.7)
NSTE-ACS	99	(37.2)
Non-ACS	72	(27.1)
Clinical outcomes at 30 days, *n* (%)		
Deceased	0	(0)
New heart failure	1	(0.4)
New myocardial infarction	0	(0)
New stent thrombosis	0	(0)
New stroke	0	(0)
New bleeding event	37	(14.0)
Unplanned cardiac rehospitalisation	3	(1.1)
PCI-specific PROM, mean (SD)		
Overall score	7.6	(2.0)

*STEMI* ST-Elevation Myocardial Infarction, *NSTE-ACS* Non-ST-segment-elevation acute coronary syndrome, *Non-ACS* Non-acute coronary syndrome, *CABG* Coronary artery bypass grafting, *PCI* Percutaneous Coronary Intervention, *PROM* Patient Reported Outcome Measure

For the second task, participants were provided with a list of proposed endpoints (e.g. all-cause mortality at 6 months, major adverse cardiac and cerebrovascular events (MAACE) at 12 months), identified from the literature and clinical experts, that could be used to validate the composite measure to predict patient recovery post PCI in future research. Participants were asked to rank from 1 (most important) to 8 (least important) the suggested endpoints and nominate any additional endpoints to consider. The first round survey was open from the 22nd of November 2017 to the 3rd of December 2017.

The second round of the Delphi invited the same experts from Round 1 to participate. Participants were presented with the median scores and summarised rationales for each of the clinical outcomes rated in Round 1. Based on this information, participants were asked to re-rate each of the clinical outcomes. Participants were also presented for the first time with the findings from the analysis of patient data (MC-PROM and clinical outcomes) from study 1, and asked about inclusion of potentially correlated clinical outcomes. They were then asked to assign a percentage weight for each of the proposed clinical outcomes and the MC-PROM summary score for inclusion in the composite measure. The weightings for all the outcomes needed to add up to 100%. The second round survey was open from the 26th of April to the 8th of June 2018.

The third and final round of the Delphi invited the same experts to participate. Participants were presented with the aggregated results of the Round 2 weightings, two proposed composite measure models, as well as examples of how the composite measure would be calculated. The third round survey was open from the 28th of June 2018 to the 18th of the July 2018.

#### Data analysis

At the end of the round one Delphi survey, the median and interquartile range of the rating of importance for each of the seven clinical outcomes was calculated. Free text comments in relation to the rationale for prioritisation of each of the clinical outcomes were summarised thematically. The mean and median rankings of suggested endpoints for the validation of the composite measure was also calculated.

For round two of the Delphi survey, the median and interquartile range of importance rating for each clinical outcome was recalculated. Additionally, the findings from the analysis of the patient outcomes from component 1 were summarised. Thirdly, the median of the proposed weightings were calculated for the clinical outcomes and the MC-PROM data. For the weightings, the median was recalculated so that the sum of the outcomes equalled 100.

At the conclusion of the third round, the data analysis allowed the creation of a model of a single composite measure that included both prioritised clinical outcomes and the MC-PROM, weighted by the expert panel as to each outcomes relative importance. All statistical analyses were conducted using SPSS Version 25.0 (IBM Corp, IBM Statistics for Windows. Armonk, NY: IBM Corp).

#### Scoring the composite measure

**Fig. 3:**
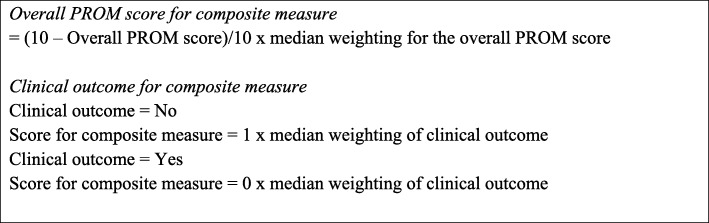
Composite Measure Calculation

After the creation of a composite measure model, a scoring framework was developed. Positive scoring was used so that a higher score reflected a better patient outcome. Therefore, if an identified (adverse) clinical outcome occurred, a zero was assigned; and if an identified clinical outcome was not present, a ‘one’ was assigned. The clinical outcome was then multiplied by the median weighting to create its component of the composite measure. Box 1 outlines the calculation for including the overall MC-PROM score and the individual clinical outcomes in the composite measure. The weighted scores for the MC-PROM and each clinical outcome were summed to provide an overall composite score. The composite measure is scored out of 100. A higher score indicates better health and wellbeing for the patient; a lower score indicates poorer health and wellbeing for the patient (Fig. 3).

Ethics approval for this project was obtained from Monash University Human Research Ethics Committee and the respective hospital ethics committees.

## Results

### Study 1:factor analysis of MC-PROM and clinical outcomes routinely collected in VCOR

#### Participants

The MC-PROM was administered to 266 patients who had undergone a PCI at one of the participating hospitals during their routine 30-day follow-up phone call with VCOR. No patient declined to complete the MC-PROM. Table 1 presents the patient characteristics including the clinical outcomes and mean overall MC-PROM score. The composition of our study sample was broadly similar to the overall VCOR patient cohort and with the British NICOR-BCIS Audit of Percutaneous Coronary Interventions [24] . There was a predominance of males (80% our study, 76% VCOR cohort), with similar mean ages (64 years our study, 66 years VCOR cohort). However, there were some differences in relation to clinical presentation between the two groups. In our study, 73% of patients presented with an ACS, with a near-even split between acute ST elevation myocardial infarction (STEMI) (36%) and non-ST elevation ACS (37%). The VCOR cohort had a lower proportion of patients with ACS (53%), 21% with STEMI and 30% with non-ST elevation ACS [25]. None of our participants died at 30 days, and only 16% of participants (*n = 41)* reported experiencing a clinical outcome post-procedure. This is consistent with the overall VCOR patient cohort where the rates of any of these outcomes range from 0.4 to 13.7% [25].

#### Exploratory factor analysis

The clinical outcomes and MC-PROM data were subjected to principal component analysis. Prior to performing the principal component analysis, the suitability of data for factor analysis was assessed. Inspection of the correlation matrix (Table 2) revealed that only two outcomes, unplanned cardiac rehospitalisation and new heart failure within 30 days following PCI, demonstrated a moderate association (*r* = 0.58). A factor analysis was therefore not appropriate, and factor loadings were not obtained.

**Table 2:** Correlation matrix for clinical outcomes and PCI-specific PROM

	Deceased	New heart failure	New myocardial infarction	New stent thrombosis	New bleeding	New stroke	Unplanned cardiac rehospitalisation	Overall PROM score
Deceased	1.00	–	–	–	–	–	–	–
New heart failure	–	1.00	–	–	−0.03	–	0.58	0.04
New myocardial infarction	–	–	1.00	–	–	–	–	–
New stent thrombosis	–	–	–	1.00	–	–	–	–
New bleeding	–	−0.03	–	–	1.00	–	0.06	0.02
New stroke	–	–	–	–	–	1.00	–	–
Unplanned cardiac rehospitalisation	–	0.58	–	–	0.06	–	1.00	0.10
Overall PROM score	–	0.04	–	–	0.02	–	0.10	1.00

Weak associations were observed between the overall MC-PROM score and new heart failure (*r* = 0.04), new bleeding event (*r* = 0.02) and unplanned cardiac rehospitalisation (*r* = 0.10). Further analysis with Mann-Whitney U tests, however, revealed no significant differences between the overall MC-PROM median scores and the clinical outcomes at 30 days post-procedure. The potential correlation between unplanned cardiac rehospitalisation and new heart failure was included as a choice question in Round 2 of the Delphi survey in order to determine clinical experts’ opinion about which outcome should be included in the composite measure.

### Study 2: modified Delphi to determine the weighting of the factors in the composite measure

#### Participants

Thirty-two clinical experts were invited to participate in the Delphi surveys. The response rate for the first round was 56% with 18 clinical experts completing the survey. Invitations to participate in the later survey rounds were only sent to the 18 clinical experts who participated in round one. The response rate for round two and three were 44% (*n* = 8) and 38% (*n* = 7) respectively.

##### Delphi expert survey

***Round 1.***

*Rating, inclusion and weighting of individual measures in the composite measure* The highest rated MC-PROM item was ‘Pain or discomfort on exertion at 30 days post-PCI’ (median = 9), and the lowest was ‘Trouble falling asleep or staying asleep at 30 days post-PCI’ (median = 5). Examples of rationales regarding the highest and lowest rated MC-PROM items include, respectively, *“Chest pain and shortness of breath are key symptoms that are hoped to be improved with stents”* and *“Many patients have sleep problems!”* The highest rated clinical outcome was ‘new stent thrombosis within 30 days of PCI’ (median rating = 9), and the lowest was ‘new heart failure within 30 days of PCI’ (median = 6). Examples of rationales regarding the highest and lowest rated clinical outcome components include, respectively, *“Very important as stent thrombosis is often due to technical PCI factors or stent design so very clearly a marker of PCI quality”* and *“PCI not directly related to heart failure occurrence - it can still occur even with successful PCI”*.

*Proposed endpoints for composite measure validation.*

The highest rated endpoint was ‘mortality at 6 months’ (median ranking of 2) and the lowest rated endpoint was ‘rehospitalisation (any) at 6 months’ (median ranking of 7)**.** Examples of rationales regarding the highest and lowest ranked proposed endpoints include, respectively, *“Positive benefit on total mortality or MACCE at one year seems a very hard endpoint”* and *“not a great believer in re-hospitalisation as a hard endpoint that is very relevant clinically”.* The first six months post PCI were considered an important endpoint for PCI related adverse events: *“First six months is critical post PCI re adverse events. Beyond this other co morbidity factors may well have greater significance. It is important to appreciate both.”* Other endpoints proposed by the expert panel included: return to employment at six and 12 months; number of subsequent cardiac procedures; quality of life (QoL) assessment; aspirin and statin prescription at 30 days; new diagnosis of depression or anxiety; and, risk-adjusted mortality.

***Round 2.***

**Fig. 4:**
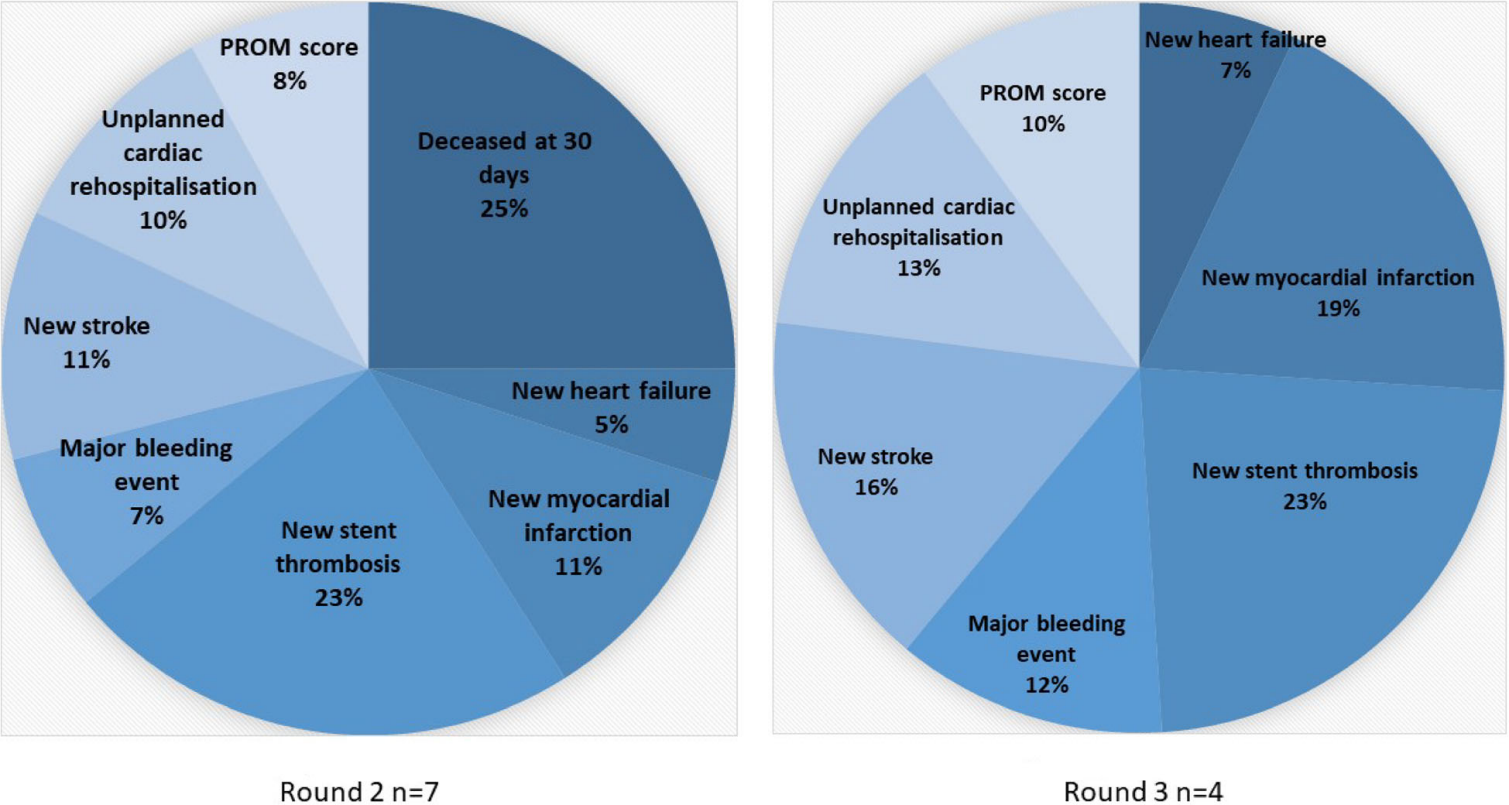
Weighting of clinical outcomes and PROM score by clinical experts in round 2 and 3 of Delphi study

In Round 2, the highest rated clinical outcome was again ‘new stent thrombosis within 30 days of PCI’ (median rating = 9), and the lowest was again ‘new heart failure within 30 days of PCI’ (median = 6). Following the presentation of the clinical outcome correlation results, four of the respondents chose the option to ‘*include unplanned cardiac rehospitalisation only’* in the composite measure, while four respondents chose the option to *‘include both unplanned cardiac rehospitalisation and new heart failure’*. No respondents chose the option to ‘*include new heart failure only’*. The MC-PROM score weighting ranged from 5 to 25%. The weightings of the clinical outcomes and MC-PROM score are presented in Fig. 4.

*The inclusion or exclusion of ‘deceased at 30 days’ from the composite measure.*

When calculating the composite score, the issue of whether or not to include or exclude ‘deceased at 30 days’ was raised by investigators and the clinical experts. The rationale for each argument is presented below:

1. **Inclusion of ‘deceased at 30 days’**: The median weighting for ‘deceased within 30 days’ was 23% (rating range 15–50%). None of the clinical experts gave this indicator a weighting of zero or 100%. Therefore it should be included in the composite measure.
2. **Exclusion of ‘deceased at 30 days’:** Using the proposed formula to calculate the composite measure, a patient can be deceased within 30 days, yet be able to achieve a relatively good composite score. If the patient is deceased, they are unable to complete the MC-PROM and therefore this score is not able to be included in the composite measure. Death is the ultimate negative outcome and therefore if a patient dies a zero should be awarded for the composite measure.

The rationales above and example patient scenarios were developed and presented to the clinical experts in round 3.

***Round 3.***

In Round 3, the adjusted median of the weighted individual clinical outcomes from Round 2 of the Delphi were presented. Examples of patient scenarios were presented with the calculated composite score (Table 3) and the rationales for including and excluding ‘deceased at 30 days’. Two out of seven participants chose to include ‘deceased at 30 days’ and five participants chose to exclude ‘deceased at 30 days’ in the final composite measure. Four of the five clinical experts re-weighted the MC-PROM score and clinical outcomes based on removing ‘deceased at 30 days’. The highest weighting was given to ‘new stent thrombosis within 30 days of PCI’ (23%), and the lowest to ‘new heart failure within 30 days of PCI’ (7%), while the ‘MC-PROM score’ received a weighting of 10%. ‘Deceased at 30 days’ was excluded from the composite measure and the new weightings applied. Example patient scenarios were calculated to demonstrate the composite measure scores across different clinical outcomes and overall MC-PROM scores (Table 3).

**Table 3:** Composite measure score calculations based on patient scenarios

	Patient 1 had an uneventful post-PCI recovery period, and scored a PROM of 2.		Patient 2 developed a new stent thrombosis on Day 17 post-PCI. Nevertheless, Patient 2 felt unwell in the 7 days leading up to the 30 day post PCI call and scored a low PROM of 7.		Patient 4 had a new myocardial infarction on Day 17 post-PCI and died on day 20 post-PCI	
Individual composite measure and (%) weighting	Status	Calculations	Status	Calculations	Status	Calculations
New heart failure within 30 days post-PCI (7)	No (1)	7 × 1 = 7	No (1)	7 × 1 = 7	No (1)	7 × 1 = 7
New myocardial infarction within 30 days post-PCI (19)	No (1)	19 × 1 = 19	Yes (0)	19 × 1 = 19	Yes (0)	19 × 0 = 0
New stent thrombosis within 30 days post-PCI (23)	No (1)	23 × 1 = 23	Yes (0)	23 × 0 = 0	No (1)	23 × 1 = 23
Major bleeding event within 30 days post-PCI (12)	No (1)	12 × 1 = 12	No (1)	12 × 1 = 12	No (1)	12 × 1 = 12
New stroke within 30 days post-PCI (16)	No (1)	16 × 1 = 16	No (1)	16 × 1 = 16	No (1)	16 × 1 = 16
Unplanned cardiac rehospitalisation within 30 days post-PCI (13)	No (1)	13 × 1 = 13	No (1)	13 × 1 = 13	No (1)	13 × 1 = 13
Overall PROM score (10)	2	10–2 = 8	7	10–7 = 3	Cannot be administered	
Composite measure score (out of 100)		98		70		N/A

## Discussion

In this study, we have developed a composite measure that includes a PROM designed specifically for patients following a PCI (MC-PROM) with objective clinical outcomes. The implementation of PROMs in healthcare to promote patient-centred care has been recognised as an international priority [3, 26]. However, the subjective nature of PROMs does not always correspond to the clinical outcome of the patient, while the objective nature of the clinical outcome measures may not identify important barriers to patient recovery (e.g. confidence in carrying out usual tasks). Additionally, through electronic administrative data, audits and registries, a vast amount of clinical outcome measures are available which can be difficult to understand. A composite measure can condense a number of outcome measures into a single score which can simplify the data and provide an overview of patient recovery and quality of care [27]. Combining PROMs with objective clinical outcomes into a single composite measure ensures that both the clinician and patient perspectives are captured, providing an overview of patient recovery and quality of care [27].

The relatively low final weighting of the MC-PROM suggests that there may be an acceptability issue for clinicians in relation to the use of a PROM as an outcome measure, which is not uncommon [28, 29]. However, it should be noted that some of the expert panel did weight the MC-PROM at 25% of the composite measure score. This indicates varying understanding and engagement with the concept of PROMs by the panel members. The aim of the MC-PROM was to provide an indication of recovery for the patient in the context of a CQR. Given that the vast majority of patients with PCI do not experience a significant adverse clinical event, the use of adverse outcomes as key clinical outcome measures by VCOR does not reflect the experience and recovery of the majority of the registry population. Further, experiences that many patients encounter post-PCI procedure such as lack of confidence, anxiety/depression, and ongoing mild clinical symptoms, may not be recognised or appropriately managed when they are not incorporated within routine outcome measurement. This composite measure approach seeks to provide a more meaningful outcome measure for a majority of patients during early recovery following their procedure. With increasing use, and an increasing emphasis on PROMs in CQRs [4], the acceptability of the MC-PROM in the composite measure may increase.

A strength of this study was that the composite measure was developed using a multi-method approach that included a factor analysis to determine the clinical PCI outcomes that can be combined with the MC-PROM. We then obtained a clinically meaningful weighting of the clinical outcomes and PROM score identified in Study 1 by undertaking three sequential Delphi surveys with a recognised group of clinical experts. Thus, we were able to develop a composite measure for patients following PCI that combines clinically important indicators as well as patient’s perspective of recovery. This study represents methods that can be replicated in other CQRs involving different patient populations and interventions to develop a composite measure. Additionally, by involving clinical and registry experts, as well as patients, to actively participate in the development of the PROM and composite measure, there is validity and acceptability for the final composite measure. Future validation studies of the PROM and engagement with clinicians will be important to champion the use of the PROM in the CQR and as part of a composite measure. The endpoints proposed by the clinical experts can also be used in future validation studies.

There are a number of limitations that need noting. Firstly, there was a decrease in expert panel participation from Round 1 to 3 (from 18 to 7 survey completions). However, there was little change in the ratings and weightings of the individual clinical outcome measures, and the weighting of the PROM score, between rounds. We were also unable to complete a factor analysis and obtain factor loadings because only two outcomes demonstrated a moderate association. This is likely due to the low number of participants experiencing a cardiac event post intervention. Given the low prevalence rates of these clinical outcomes post-PCI, a larger sample should be considered when planning future validation studies of the composite measure. Nevertheless, it is important to keep in mind that the prevalence rates observed in this study is consistent with rates reported in VCOR [25] as well as previous literature [30, 31]. Finally, patients were not involved in the development of this composite measure and did not have an opportunity to weight the PROM score with the clinical outcomes. This composite measure is designed to be used by clinicians and hence we focused on obtaining their input. However it is important to note that patients who have had a PCI were involved in each stage of the development of the MC-PROM [19–21]**.**

## Conclusions

In this study we developed a composite measure that combined the MC-PROM designed specifically for patients following a PCI with clinical outcomes to assess recovery at 30 days. The MC-PROM was weighted at 10% of the overall composite measure with clinical outcomes comprising the rest of the score. Utilising PROMs in clinical registries to assess quality of care is gaining traction internationally. Combining a PROM with clinical outcomes to provide a composite measure of quality of care in PCI is particularly important where adverse clinical events (which are traditional clinical outcome measures) are rare and may not be appropriate for assessing quality of care for a majority of patients.

## Data Availability

The datasets generated and/or analysed during the current study are not publicly available as we have ethical and legal restrictions on sharing a de-identified dataset because we did not seek approval from study participants to have data shared publicly. Please contact the Executive Officer at Monash University Human Research Ethics (MUHREC project 2016–1409) for any data requests.

## Abbreviations

ACS: Acute coronary syndrome
CABG: Coronary Artery Bypass Graft
CQR: Clinical quality registries
MAACE: Major adverse cardiac and cerebrovascular events
MC-PROM: Monash University Cardiac PROM
PCI: Percutaneous coronary intervention
PROM: Patient reported outcome measure
PROMs: Patient reported outcome measures
QoL: Quality of life
STEMI: ST elevation myocardial infarction
VCOR: Victorian Cardiac Outcomes Registry
